# Inhibition of UHRF1 Improves Motor Function in Mice with Spinal Cord Injury

**DOI:** 10.1007/s10571-024-01474-5

**Published:** 2024-04-22

**Authors:** Shuai Cheng, Hui Guo, Mingyu Bai, Yang Cui, He Tian, Xifan Mei

**Affiliations:** 1 School of Basic Medicine, Jinzhou Medical University, Jinzhou, China; 2 Liaoning Provincial Collaborative Innovation Center for Medical Testing and Drug Research, Jinzhou Medical University, Jinzhou, Liaoning China; 3 Jinzhou Medical University, Jinzhou, China; 4 Jinzhou Medical University, Linghe District, No. 40, Section 3, Songpo Road, Jinzhou, Liaoning Province China

**Keywords:** Spinal-cord injury, Microenvironment, Molecular regulation, UHRF1, Immunity

## Abstract

Spinal-cord injury (SCI) is a severe condition that can lead to limb paralysis and motor dysfunction, and its pathogenesis is not fully understood. The objective of this study was to characterize the differential gene expression and molecular mechanisms in the spinal cord of mice three days after spinal cord injury. By analyzing RNA sequencing data, we identified differentially expressed genes and discovered that the immune system and various metabolic processes play crucial roles in SCI. Additionally, we identified UHRF1 as a key gene that plays a significant role in SCI and found that SCI can be improved by suppressing UHRF1. These findings provide important insights into the molecular mechanisms of SCI and identify potential therapeutic targets that could greatly contribute to the development of new treatment strategies for SCI.

**Graphical Abstract:**

Downregulation of UHRF1 promotes the recovery of motor function in mice with spinal cord injury. By analyzing the RNA sequencing results of mice with spinal cord injury for 3 days and selecting UHRF1 for experimental validation, the study found that inhibiting UHRF1 can promote motor function recovery.

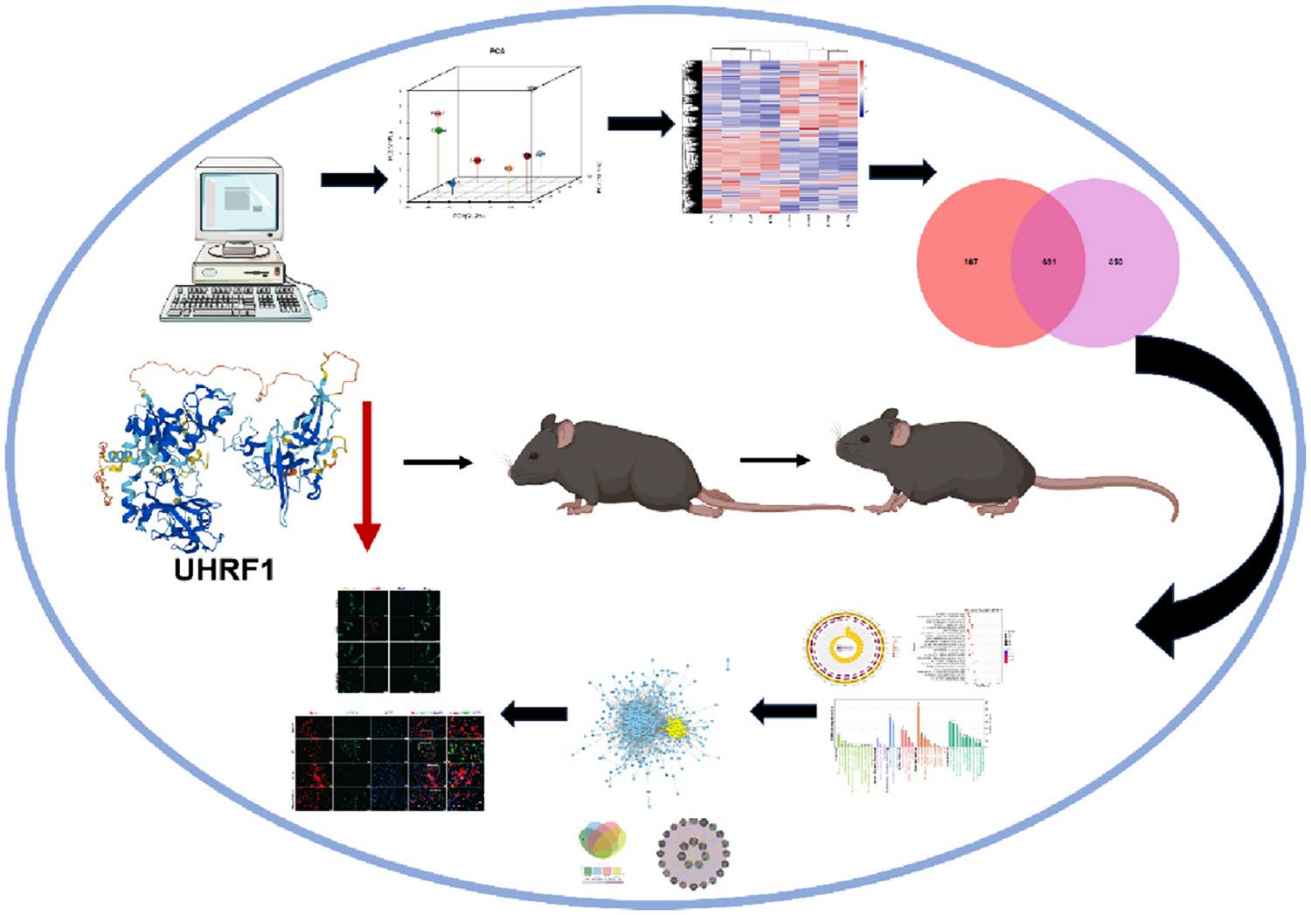

## Introduction

As a severe traumatic injury to the central nervous system (McDonald & Sadowsky 2002), the society, familial, and individual burden resulting from spinal cord injury (SCI) is immense and incalculable. (Wang et al. 2020a, b). The inflammatory response in the acute stage, scar formation in the subacute stage, and nerve regeneration and functional reconstruction in the chronic stage after SCI are crucial to the prognosis of the disease (Zhou et al. 2022). With the continuous understanding of the disease process of SCI, intervention in the early stage of the disease is becoming increasingly important (Anderson et al. 2018; Kathe et al. 2022). In addition, controlling inflammation during the preoperative period and promoting circulation to the site of injury is pivotal for recovery. However, the condition of most SCI patients is very complex, and single treatment methods cannot restore the original neurological function, leading to a high probability of high-level paraplegia or lower limb paralysis (van der Vlist et al. 2022).

Currently, there are numerous studies on the exploration of neural repair mechanisms after SCI, involving gene regulation, molecular docking, protein interactions, cell communication, and organ dialogue (Kirshblum et al. 2016). The ultimate goal of these complex repair and regulation mechanisms is to promote nerve cell regeneration, control neuropathic pain, and restore motor function (Bauman et al. 2017). Therefore, our team has focused on the repair of neuronal cells after SCI and the restoration of motor function in C57 mice, including the control of inflammation after injury (Xu et al. 2022a, b), the control of oxidative stress, the phenotype transformation of microglia (Zhu et al. 2017), the regulation of glucose metabolism to facilitate energy production in the injury microenvironment, regulation of iron death, and regulation of autophagy (Xu et al. 2022a, b). Through numerous explorations, we have become increasingly concerned with changes in the early microenvironment after SCI and the mutual regulation among various molecules. Actively searching for new therapeutic targets between molecules is of great significance for the early recovery of SCI (Qu et al. 2023).

Bioinformatics analysis is a method that can reduce the number of experimental animals and identify a large number of different factors that occur after disease onset (Morrissey et al. 2017), which can lead to the discovery of new pathways and mechanisms for disease recovery or other effects (del Val et al. 2004). It has positive implications for medical research (Wang et al. 2015). Using bioinformatics methods, the diverse modifications in factors following the onset of disease can be interpreted, analyzed, and further explored (Wang, Liu, Xie, Cai, & Xu 2020a, b). The sequencing technologies of the second and third generations have rapidly developed, global research teams focus on different aspects of the same disease for sequencing and exploration. The sequencing data are freely available and shared, which can further save time for data collection, analysis, and interpretation (Luo et al. 2020).As the second most abundant trace element in the human body, zinc has positive implications for recovery after spinal cord injury. We found that intraperitoneal injection of zinc gluconate can improve the motor function of mice after spinal cord injury (Li et al. 2020). However, we are still exploring which molecular targets of zinc play a role in the spinal cord.

In summary, we decided to mine the sequencing data of spinal cord injury in existing public databases. It is hoped that these data will identify new therapeutic targets for spinal cord injury. We will perform basic experimental validation of the targets we discover to confirm their presence and changes in animals and cells. Therefore, in the following story, we will use bioinformatics analysis combined with experimental validation to explain our findings and explore new targets for recovery of motor function in mice after spinal cord injury.

## Materials and Methods

### Animal Selection and Model Preparation

The C57BL/6 mice used in this study (half male and female, weighing 25–30 g, 6–8 weeks old) were purchased from Liaoning Changsheng Biotechnology Co.Ltd. (Benxi, China; license number: SCXK (Liao) 2020–0001). Experimental mice were housed in standard cages (5 per cage) in a specific pathogen-free laboratory center of Jinzhou Medical University, with a 12-h/12-h light–dark cycle. Mice had free access to food and water, and the ambient temperature was maintained at 22–24 °C. In addition, let the mice acclimate to the environment for one week before the official start of the animal experiment. All surgeries were approved by the Animal Ethics Committee of Jinzhou Medical University (approval number: SYXK (Liao) 2019–0007).

According to previous reports, mice were anesthetized with 1% sodium pentobarbital. The hair on the back was removed, disinfected with povidone iodine, and each mouse was placed in a prone position on the operating table with the spine fully exposed, and then a laminectomy was performed on the T9/T10 vertebral body. The spinal cord was completely exposed, and IH-0400 (Infinite Horizon Impactor) was used to uniformly impact the spinal cord at T9/T10 at 50kdyn. In the sham operation group, laminectomy was performed without spinal cord contusion, and the skin was sutured after hemostasis. Throughout the experiment, mice were observed daily for health status, cage activity, and infection. Manual bladder massage was performed twice a day for urination in the first week after surgery, and once a day from the second week until the mice regained spontaneous urination. Two hours after surgery, mice were intraperitoneally injected with zinc gluconate (30 mg/kg) or normal saline (0.1 ml) for 3 days. The inhibitor is diluted and injected intraperitoneally (injected simultaneously with zinc gluconate) according to the dosage recommended by the company where the reagent was purchased (Shan Wen1, 2023).

### Cell Culture and Model Construction

For in vitro experiments, we selected PC12 cells (Cat#ab279978), which are common in nervous system diseases, and purchased them from Abcam. All cells were cultured in a pre-set cell incubator at 37 °C, 5% CO2, and digested with 0.25% trypsin (Bioind) during cell passaging. The medium used was Dulbecco's modified Eagle medium (Gibco), containing fetal bovine serum (10%), penicillin (100 U/mL) and streptomycin (100 μg/mL).

For oxygen and glucose deprivation (OGD), select PC12 cells at an appropriate density, and change their medium to phosphate-buffered saline (PBS, pH 7.4). Then nitrogen gas (20 L/min, 5 min) was filled in the hypoxic incubator to keep the oxygen concentration below 1%. After 2 h of hypoxia and glucose deprivation, cells were removed from the hypoxic incubator and cultured in normal growth medium for 1 day. The control group was incubated normally.

### Immunofluorescence

Mice were sacrificed with overdose of anesthesia. After perfusion, the spinal cord tissue at 0.8–1 cm around the injury site was collected, placed in a gradient sucrose solution to fully submerge, and then 10 μm frozen section was performed.

Before staining, the frozen sections of different groups were washed to remove the OCT embedding agent. After permeabilization with 0.3% Triton X-100 for 15 min, the sections were blocked with goat serum (ZSGB-BIO) for 2 h. Subsequently, serum diluted primary antibodies (anti-NeuN, Mouse, 1:1000, Abcam, ab77315; UHRF1 Antibody, Rabbit, 1:50, Affinity, DF6929) were added and incubated overnight at 4 °C. The next day, the corresponding secondary antibody (Alexa Fluor 488 goat anti-mouse IgG, Thermo Fisher Scientific, A32728; Alexa Fluor 568 goat anti-rabbit IgG, Thermo Fisher Scientific, A-11011, both 1:1000) was incubated at 37 °C 2 h. Finally, stain with DAPI staining solution (Beyotime, C1002) containing anti-fade reagent for 15 min. A coverslip was added, and images were captured using a Leica fluorescence microscope. Cell samples are processed similarly to tissues. Briefly, fix, penetrate, block, incubate primary antibody, secondary antibody, DAPI. Finally, the fluorescence intensity was observed with a Leica confocal laser scanning microscope.

### Footprint Analysis

To detect hindlimb activity in mice, we performed footprint analysis. After different treatments, the forelimbs were painted with black dye, and the hind limbs were painted with red dye. Mice were placed on absorbent paper surrounded by wooden boards and encouraged to walk in a straight line. The footprints of the mice in each group were then observed on paper.

### Basso Mouse Scale

Behavior was assessed using the Basso Mouse Scale (BMS). Three experimenters evaluated mice in a double-blind fashion on days 1, 3, 5, 7, 10, 14, and 28 postoperatively. The BMS score ranges from 0 to 9, where 0 represents no movement of the ankle joint (complete loss of movement of the hind limb) and 9 represents complete normal movement.

### Heat map, Volcano Map, GO Enrichment, KEGG Enrichment, Reactome Enrichment, Protein Interaction

Use the GEO query, limma, affy, pheatmap, and ggplot2 data packages in R language to perform data dimensionality reduction on the two data sets GSE132242 and GSE166967 (Ye et al. 2021), and then use the online tools http://www.omicshare.com , https://cn.string-db.org/, http://genemania.org/ for enrichment analysis and protein interaction prediction. Then use Cytoscope3.9.1 software for scoring screening and drawing.

### Statistical Analysis

The data analysis was performed using GraphPad prism 8.0.2. Data distribution was assessed using the Shapiro–Wilk test. For the analysis of data characterized by a normal distribution of value it. If there were more than two sets of data, one-way analysis of variance (ANOVA) was used, and a Bonferroni post hoc test was performed if the variances were equal. Mann–Whitney U-test was used if variances were unequal. BMS scores were analyzed using repeated measures and two-way ANOVA, followed by Dunnett multiple comparison test used to compare differences between groups. A p < 0.05 was considered statistically significant.

## Results

### Normalization and Principal Component Analysis of Three-Day Different Sequencing Data for Spinal Cord Injury

**Fig. 1:**
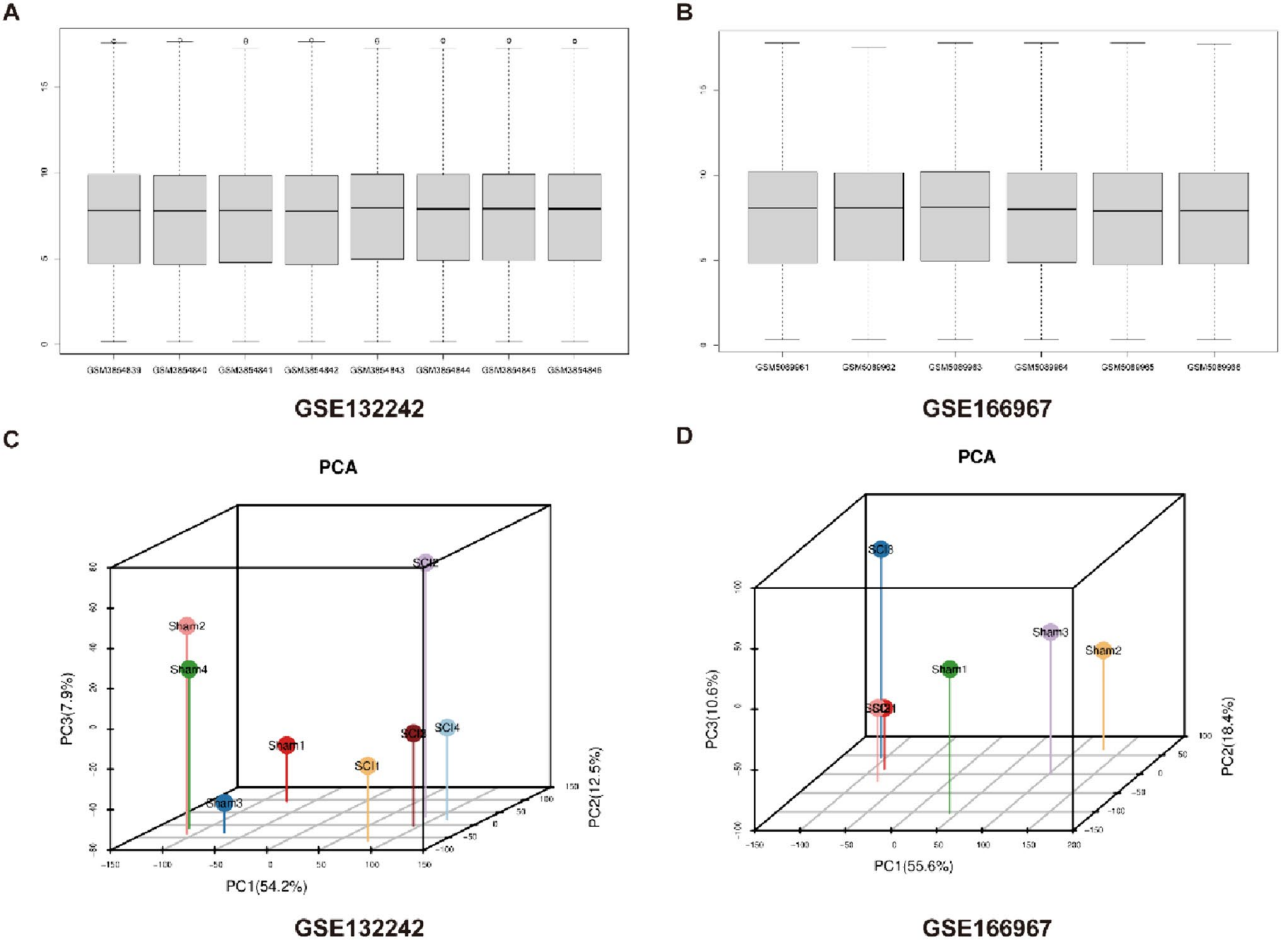
Normalization and principal component analysis of three-day different sequencing data for spinal cord injury. (**A**) Normalization of 8 samples in the GSE132242 dataset. (**B**) Normalization of 6 samples in the GSE166967 dataset. (**C**) PCA distribution plot of 8 samples in the GSE132242 dataset. (**D**) PCA distribution plot of 6 samples in the GSE166967 dataset

To identify transcriptomic changes that occur following spinal cord injury, our goal to compare transcriptomic expression profiles between spinal cord injured and healthy mice, with the goal of discovering novel targets that may contribute to explaining the pathological progression of spinal cord injury and exploring potential therapeutic interventions. Therefore, we used the two datasets GSE132242 and GSE166967 in the GEO database to obtain the original microarray sequencing data of seven mice each in the Sham group and the SCI3d group. Regarding data processing, we first use the GEO query, limma, and affy packages in the R language to obtain the original data in the data set, and then perform single-gene sequencing target correspondence, group correspondence, dimensionality reduction, and normalization processing on the original data (Fig. 1A, B). Subsequently, to verify the independence of each of the 14 samples, we used PCA analysis for data verification and found that the independence of the 14 samples in the two databases was good, and the independence and correlation between group differences met our subsequent analysis requirements (Fig. 1C, D).

### Different Genes in Mice Exhibiting Spinal Cord Injury 3 Days Old by Screening

**Fig. 2:**
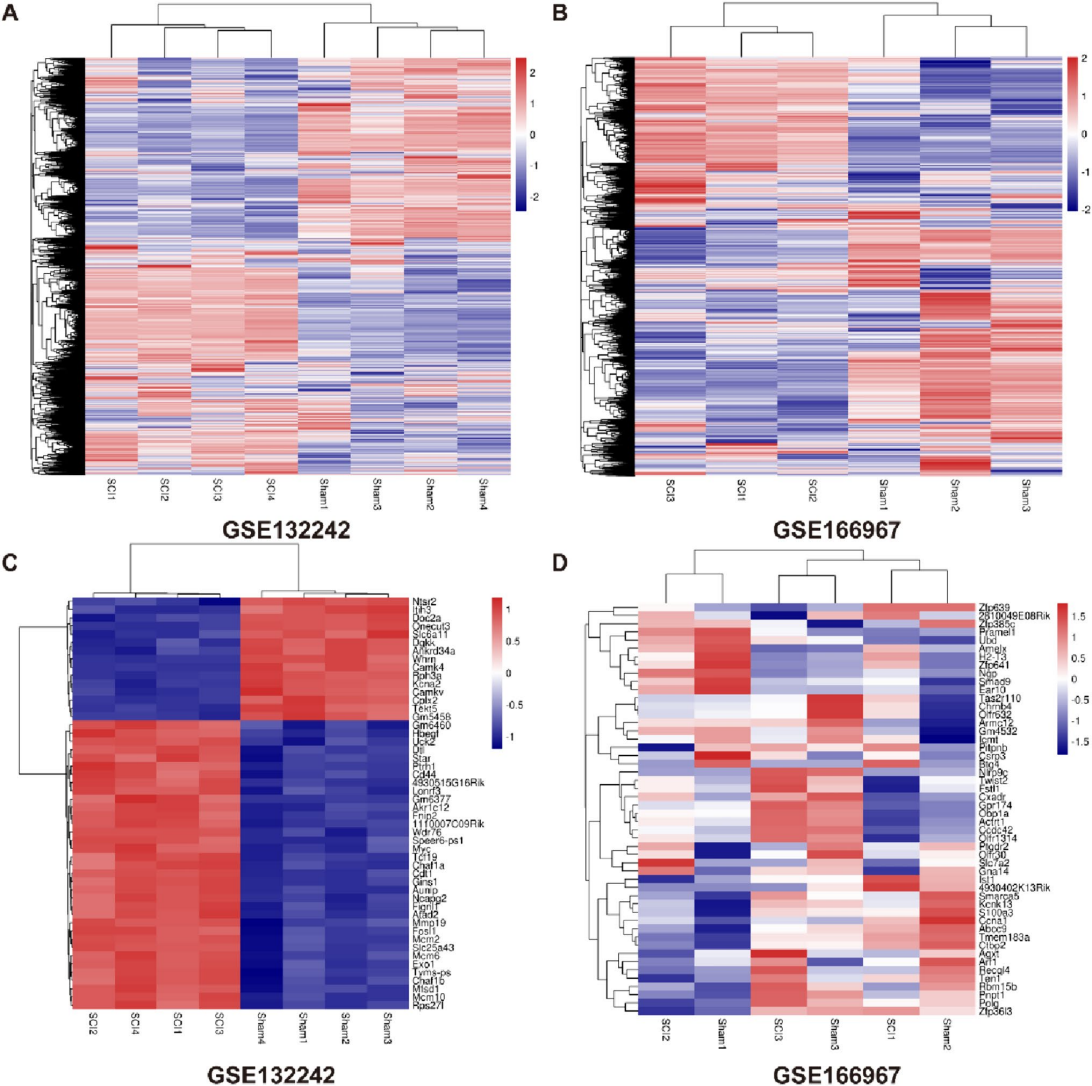
Differential genes in mice exhibiting spinal cord injury 3 days old by screening. (**A**) Gene expression heatmap of 8 samples in the GSE132242 dataset. (**B**) Gene expression heatmap of 6 samples in the GSE166967 dataset. (**C**) The heat map of the top 50 gene expression of 8 samples in the GSE132242 dataset. (**D**) The heat map of the top 50 gene expression of 6 samples in the GSE166967 dataset

Use the target mapping and the expression table corresponding to the data set to search for different genes and determine the up-regulated and down-regulated genes after 3 days of spinal cord injury for subsequent in-depth verification. Therefore, we used the pheatmap package in the R language to organize the two data sets and visualize all the different genes (Fig. 2A, B). Due to the large number of different genes in the data set, we then sorted them according to their statistical differences, using pheatmap to display the specific entries of the top 50 genes in the difference (Fig. 2C, D), some of these genes are related to inflammation, tissue fibrosis, cytokine regulation.

### The Cross-Validation of the Two DATA sets Obtained the Differential Genes with Strong Reliability

**Fig. 3:**
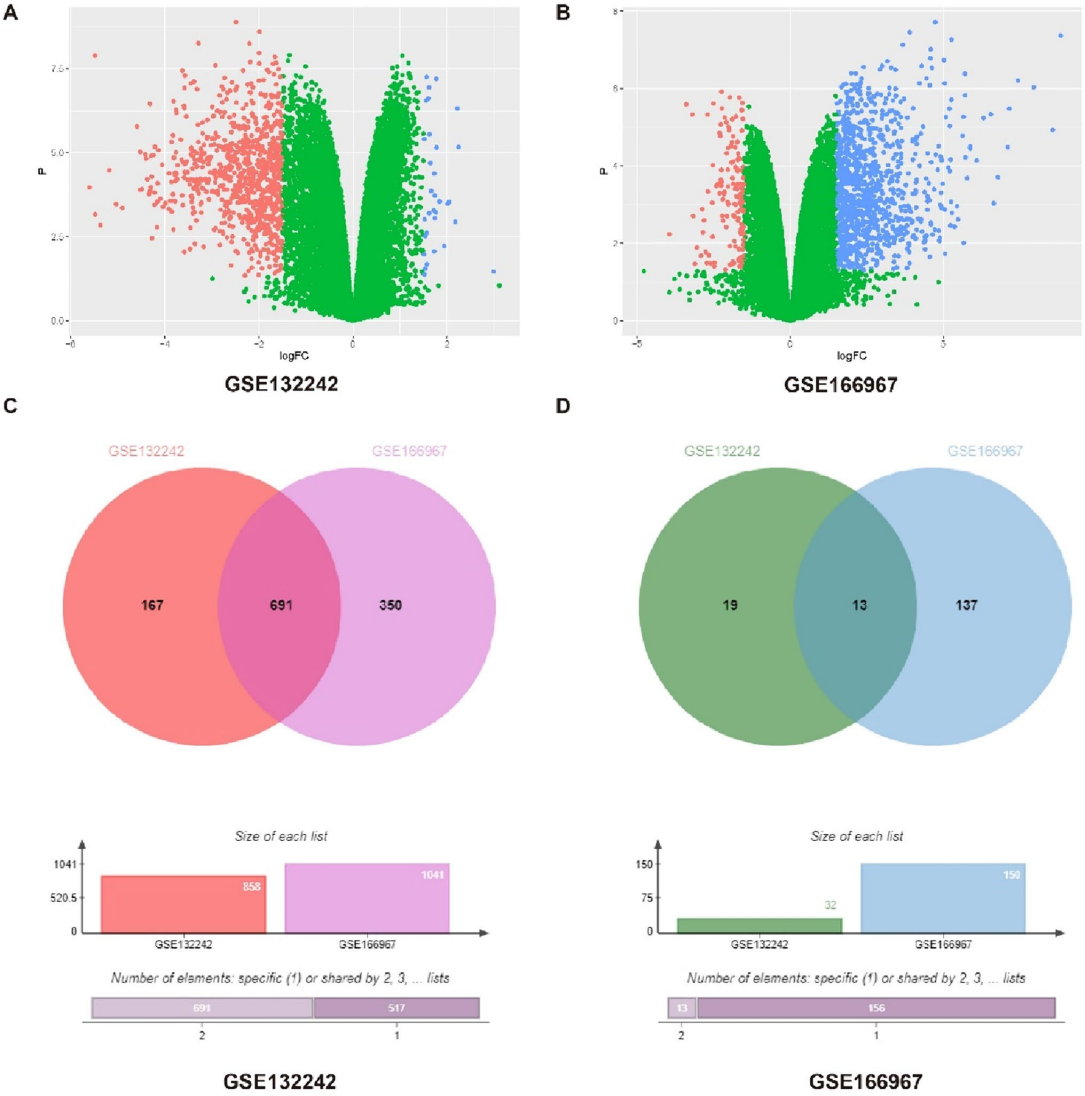
The cross-validation of the two data sets obtained the differential genes with strong reliability. (**A**) Volcano map of all differential gene expression in the GSE132242 dataset. (**B**) Volcano map of all differential gene expression in the GSE166967 dataset. (**C**) Venn diagrams of upregulated genes in GSE132242 and GSE166967 datasets. (**D**) Venn diagrams of downregulated genes in GSE132242 and GSE166967 datasets

We have analyzed the gene expression heatmap obtained by sorting the raw data. We discovered that, even though there was overlap among the factors with the most significant differences in the two datasets, they were not the same. Consequently, we opted for a strategy of mutual verification between the two separate datasets to pinpoint more dependable genes that are differentially expressed. We use the R program package ggplot2 to analyze the data set this time and consider that the condition for setting differential genes is that P. Value less than 0.05 is a differential gene, logFC greater than 1.5 is an ascending gene, and logFC is less than -1.5 is a descending gene. Generate volcano maps (Fig. 3A, B). It can be seen from the figure that in the GSE132242 dataset, 858 genes were upregulated after SCI (red), and 32 genes were downregulated after SCI (blue); In the GSE166967 dataset, 1041 genes were upregulated after SCI (blue), and SCI The post-down-regulated genes are 150 (red). Different genes obtained but it is also according to our conditional criteria. 3 days after spinal cord injury, most of the up-regulated genes, less down-regulated genes. Next, we used the Venn diagram to cross-validate the different genes in the two databases and found that there were overlapping genes in the differential genes in the two databases, which indicated that in the two different batches of sequencing, these genes were all present at 3 days after spinal cord injury Changes occur. In subsequent experiments, we will focus on the crossover genes in the two datasets (691 up-regulated genes and 13 down-regulated genes after SCI) (Fig. 3C, D).

### GO Enrichment Analysis of SCI3d Differential Genes Found that the Spinal Cord Microenvironment was Still in a State of Immune Disorder

**Fig. 4:**
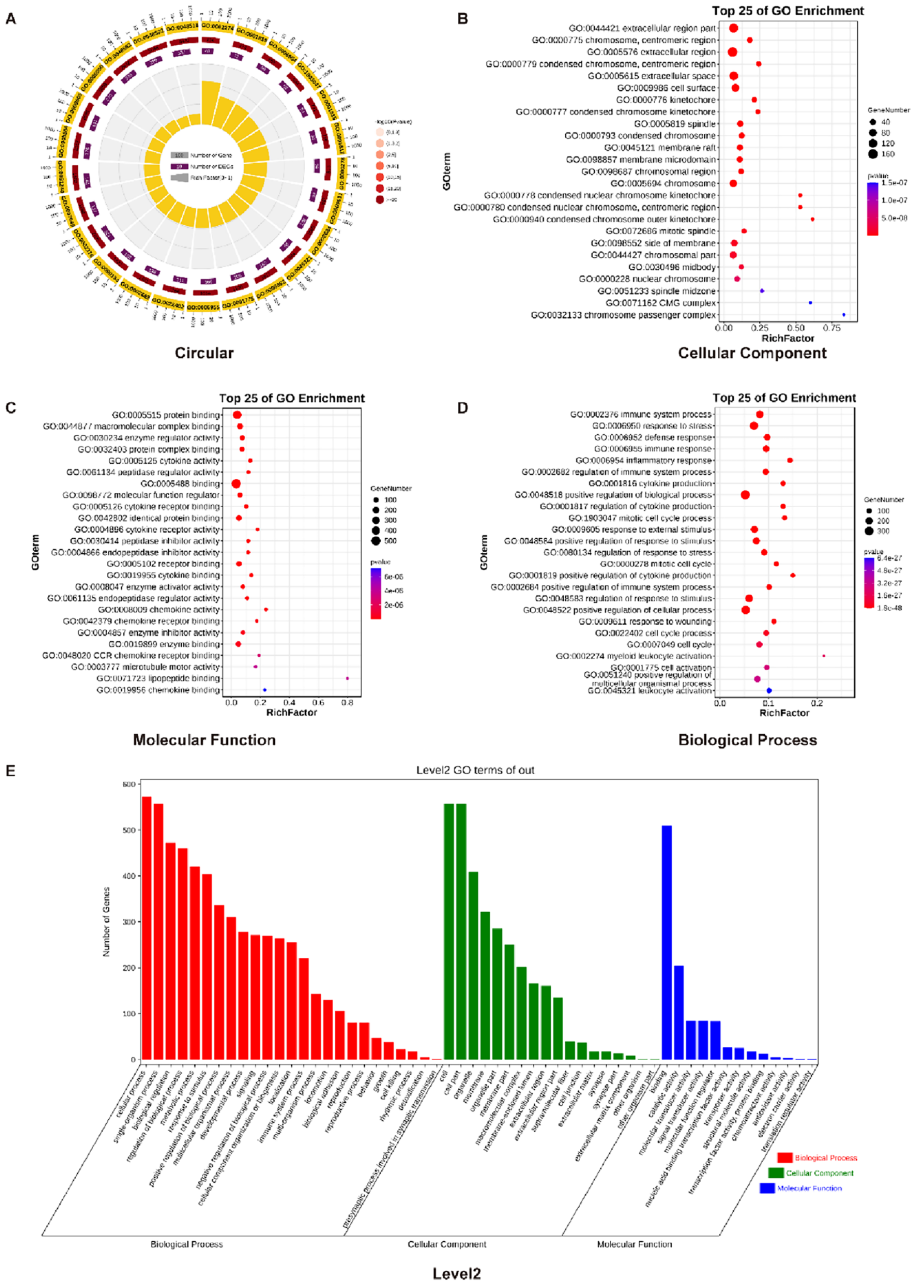
GO enrichment analysis of SCI3d differential genes found that the spinal cord microenvironment was still in a state of immune disorder. (**A**) GO enrichment analysis circle diagram of differential genes in two datasets. (**B**) GO enrichment analysis of differential genes in the two data sets Cellular ComponentTop25. (**C**) GO enrichment analysis of differential genes in the two data sets Molecular Function Top25. (**D**) GO enrichment analysis of differential genes in the two datasets Biological Process Top25. (**E**) Two-level classification diagram of GO enrichment analysis of differential genes in the two datasets

Subsequently, GO analysis was performed on the obtained data set containing 691 up-regulated genes and 13 down-regulated genes. Results revealed two highly enriched pathways: myeloid leukocyte activation (GO: 0002274) and positive regulation of cytokine production (GO:0001819) (Fig. 4A). Next, the GO enrichment results were analyzed from the aspects of cellular location (Cellular Component, CC), molecular function (Molecular Function, MF), and biological process (Biological Process, BP) (Fig. 4B, C, D). The results showed that the top three pathways with the highest enrichment of cellular component (CC) factors were all located in the extracellular region (GO: 0005576, GO:0044421, GO:0005615). The most enriched molecular function (MF) factors were interaction (GO: 0005488) and protein interaction (GO: 0005515). The most abundant biological process (BP) factors are positive regulation of biological processes (GO: 0048518), positive regulation of cellular processes (GO: 0048522), and stress response (GO: 0006950). In the secondary classification of GO, we further explored the number of differential genes in each category (Fig. 4E). Among biological processes, the highest number of enriched factors was cellular processes (GO:0009987). Among the cellular components, it is the cell (GO:0005623). Among the molecular functions, the most common is the binding activity (GO:0005488). In summary, the spinal cord injury microenvironment is in a state of severe immune disorder and the interaction and regulation of multiple factors.

### KEGG Enrichment Analysis Found that Various Chemokines and Metabolic Processes Play an Important Role in SCI3d of Spinal Cord Injury

**Fig. 5:**
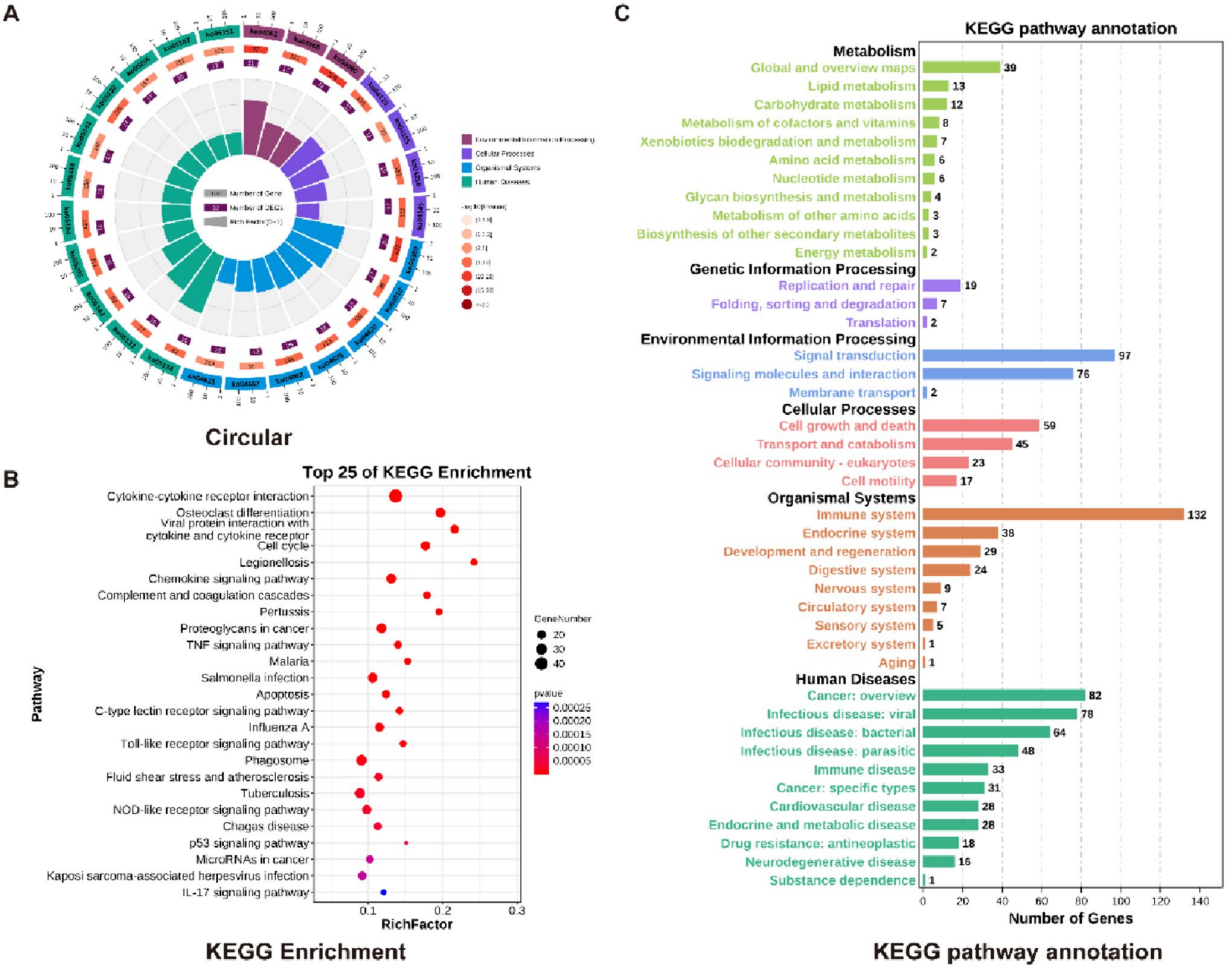
KEGG enrichment analysis found that various chemokines and metabolic processes play an important role in SCI3d of spinal cord injury. (**A**) Circle diagram of KEGG enrichment analysis of differential genes in two datasets. (**B**) KEGG enrichment analysis of top25 pathways of differential genes in the two datasets. (**C**) Pathway annotation map of KEGG enrichment analysis of differential genes in the two datasets

To comprehend the common physiological consequences exhibited by differential genes, we sought to determine whether they are concentrated and enriched in certain pathways. Therefore, we conducted the KEGG enrichment on the obtained 691 up-regulated genes and 13 down-regulated genes. KEGG enrichment results indicated that association with organic systems, cellular processes, and environmental information processing (Fig. 5A). Pathways of the TOP 5 enriched factors are cytokine-cytokine receptor interactions (42), phagosomes (28), tuberculosis (27), osteoclast differentiation (25), proteoglycans in cancer (25) (Fig. 5B). Additionally, pathways enriched in differential genes also include the classic TNF signaling pathway, Toll-like receptor signaling pathway, NOD-like receptor signaling pathway, IL-17 signaling pathway. The results of enrichment were then annotated by KEGG pathways. Three days after SCI, differential genes were enriched in six major pathways: metabolism, genetic information processing, environmental information processing, cellular processes, organismal systems, and human diseases (Fig. 5C). According to the enrichment results the KEGG, in SCI3d, inflammatory reactions and some necrotic reactions are still occurring in large quantities, abundant simultaneously, the system mobilizing numerous cell chemokines to address adverse reactions that occur and heal the damage through metabolic processes. Cells provide energy. It is noteworthy that certain factors rich in proteoglycans, lectins, phagosomes, and other pathways may serve as potential therapeutic targets.

### Reactome Enrichment Analysis Revealed the Important Role of Neutrophils in SCI3d of Spinal Cord Injury

**Fig. 6:**
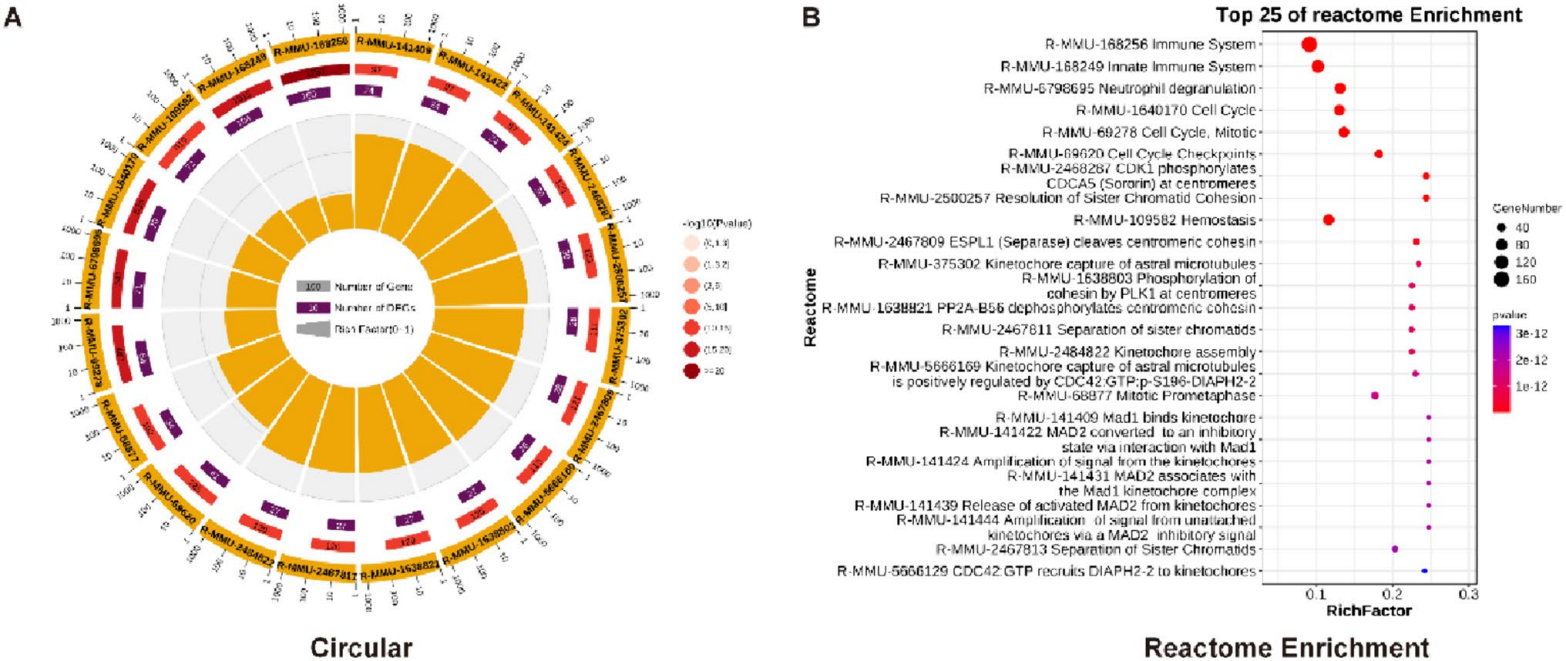
Reactome enrichment analysis revealed the important role of neutrophils in SCI3d of spinal cord injury. (**A**) Circle diagram of Reactome enrichment analysis of differential genes in two datasets. (**B**) Reactome enrichment analysis of top25 pathways of differential genes in the two datasets

To further elucidate the changes in SCI3d, we used Reactome, a clinically friendly database that has emerged in recent years, to conduct an enrichment analysis. From the Reactome enrichment analysis, we can see that the top five enriched pathways are Immune System, Innate Immune System, Neutrophil degranulation, Cell Cycle, Cell Cycle, Mitotic. Among these pathways and the enriched Top 25 pathways, the disorder of the 3D immune microenvironment of spinal cord injury and the significant of chromatin regulation at the molecular level can be confirmed again, in which many neutrophil-related factors are recruited to damaged areas to potentially against the adverse state of inflammation and gliosis scarring (Fig. 6A, B).

### The Hub Gene UHRF1 was Calculated by Differential Protein Interaction Score 3 days After Spinal Cord Injury

**Fig. 7:**
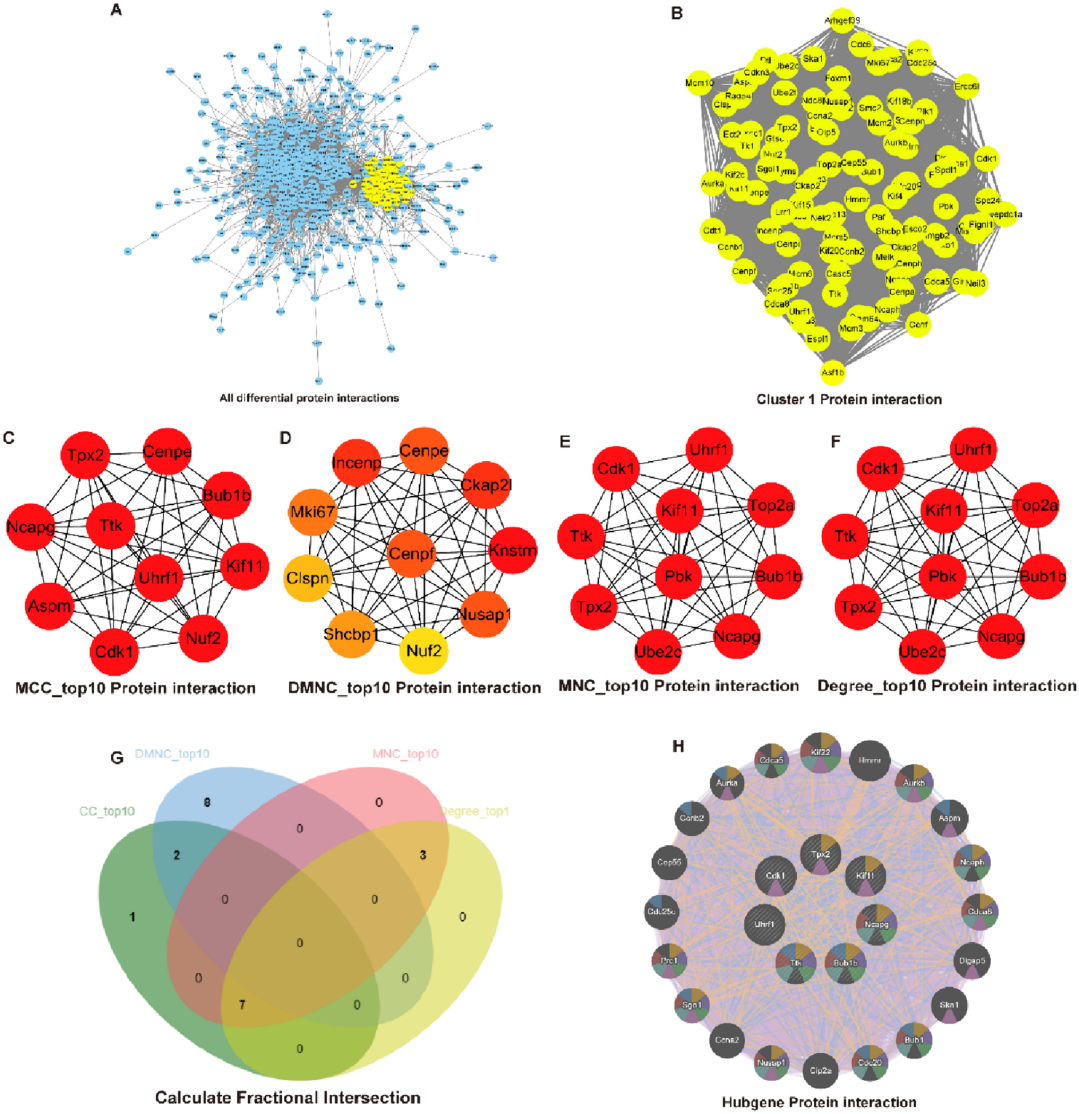
The hub gene UHRF1 was calculated by differential protein interaction score 3 days after spinal cord injury. (**A**) All differential protein interaction plots, with the group of proteins with the highest cluster score shown in yellow. (**B**) Enlarged view of the interaction of the group of proteins with the highest cluster score. (**C**–**F**) Four different algorithms MCC, DMNC, MNC, Degre calculated the TOP 10 factors for cluster 1. (**G**) Intersection of TOP 10 factors of four different algorithms. (**H**) Protein interactions predicted for the top 7 factors in the intersection set, with colors representing different biological functions

Faced with such many differential proteins, we expected to identify a subset of the most important roles among them. Therefore, we predicted the interactions of all differential factors using the online website https://cn.string-db.org/ and exported and imported the results into Cytoscape 3.9.1 for central gene screening (Fig. 7A). We first screened the clusters with the highest scores among all the differential factor networks using the MCODE plugin (yellow group in the figure), and then displayed the clusters individually (Fig. 7B), followed by scoring cluster 1 with multiple algorithms using the cytoHubba plugin, showing the TOP10 pivotal factors selected by the MCC, DMNC, MNC, Degree algorithms (Fig. 7C–F). Next, we took the intersection of multiple algorithms (Fig. 7G). Seven differential factors that appeared in all the multiple intersections were selected for subsequent study, which were Tpx2, Kif11, Ncapg, Bub1b, Ttk, Uhrf1, Cdk1, and then an online tool http://genemania.org/ was used to generate a central gene map of the TOP 7 and their interactions with the predicted proteins (Fig. 7H). In this prediction result, we found that UHRF1 seems to be different from all the other factors, he does not appear to have the same biological function as the other 6 factors, but can interact with them, so that we became interested. In summary, the seven factors with the highest calculated scores in the protein interaction network 3 days after spinal cord injury were Tpx2, Kif11, Ncapg, Bub1b, Ttk, Uhrf1, Cdk1, and that Uhrf1 may independently play other biological roles.

### Inhibition of UHRF1 Improves Motor Function in Spinal Cord Injured mice

**Fig. 8:**
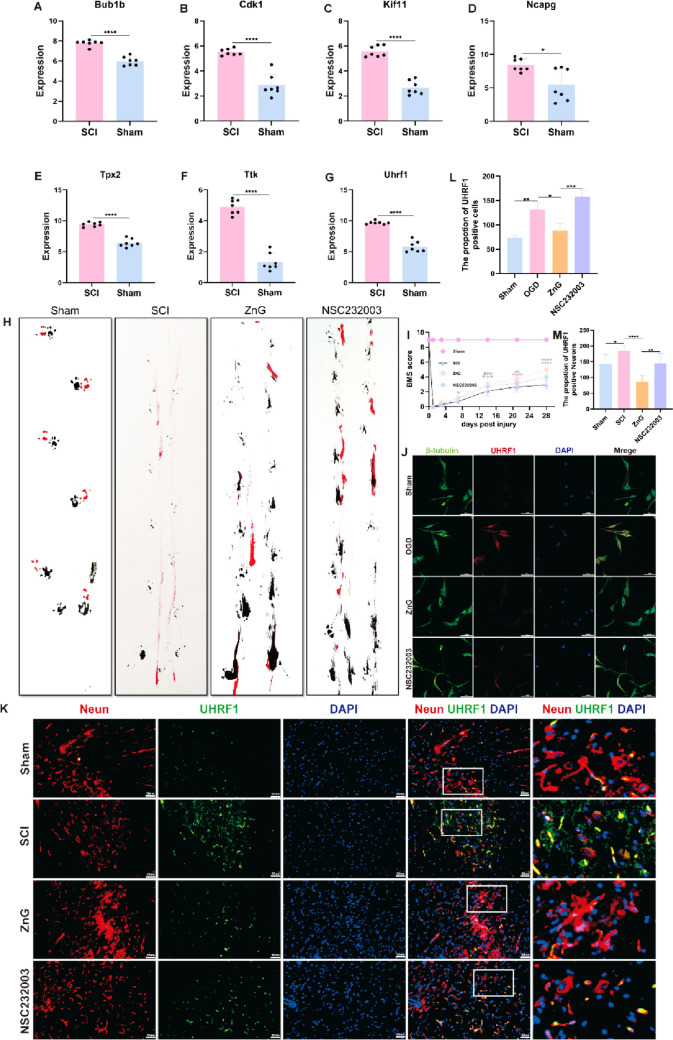
Inhibition of UHRF1 improves motor function in spinal cord injured mice. (**A**–**G**) 7 factors of Tpx2, Kif11, Ncapg, Bub1b, Ttk, Uhrf1, Cdk1 are expressed in transcriptome sequencing (*n* = 7, *P = 0.0104, **** < 0.0001). (H) Footprints of C57 mice in each group. (I) BMS scores of C57 mice in each group (*n* = 12, *P = 0.232, **P = 0.0091, ****P < 0.0001). (**J**) Immunofluorescence staining of UHRF1 in PC12 cell line. (**K**) Immunofluorescence staining of UHRF1 in C57 mouse tissues. (**L**) Immunofluorescence statistical analysis of PC12 cells in each group (*P = 0.0152, **P = 0.0027, ***P = 0.0002). (M) Immunofluorescence statistical analysis of each group of spinal cord tissue sections (*P = 0.0428, **P = 0.0040, ****P < 0.0001)

Next, we evaluated the Hub gene expression counts derived from the algorithm in the dataset (Fig. 8A–G). Further confirmation indicates that in the spinal cord injury model, the predicted mRNA expression and protein interaction scores for these seven factors are significantly different. Subsequently, we will validate the mRNA obtained from the spinal cord tissue sequencing results of C57 mice. We have chosen the specific inhibitor NSC232003 of UHRF1 and our team has been conducting intraperitoneal injection of ZnG for many years. The experimental evaluation of mouse motor function using footprint testing (Fig. 8H) and BMS score (Fig. 8I) confirmed that intervention with UHRF1 after injury improved mouse motor function. Meanwhile, we validated the expression and role of UHRF1 in neuronal cells using an in vitro neuronal cell line PC12 (Fig. 8J). We also validated the expression of UHRF1 after spinal cord injury and the role of inhibitors in mouse spinal cord tissue slices (Fig. 8K). The above experimental results indicate that three days after spinal cord injury, UHRF1 co localizes in neuronal cells. Inhibiting UHRF1 can improve spinal cord injury to a certain extent and ultimately promote the recovery of motor function in spinal cord injury mice.

## Discussion

Spinal-cord injury is a serious central nervous system injury disease, and its treatment and prognosis are difficult. Actively exploring new treatment methods and treatment targets can greatly improve the serious consequences of spinal cord injury and can also reduce the subsequent medical security burden for the society.

We expect to find targets for the treatment of spinal cord injury through various means. Thanks to the development of bioinformatics, we can more comprehensively analyze the changes of diseases at the micro level in high-throughput data to find suitable intervention methods. Therefore, as an interdisciplinary subject, bioinformatics combine knowledge from the fields of computer science, statistics, and biology (Stingele et al. 2012). With the development of science and technology, bioinformatics has played an increasingly important role in the medical field. Bioinformatics can help medical researchers conduct large-scale data analysis at the genome, transcriptome, and proteome levels(Goesmann et al. 2005) to identify genes, transcripts, and proteins associated with diseases(Manzoni et al. 2018). This helps to reveal the pathogenesis and diagnostic markers of diseases and provides a basis for disease prevention and treatment (Boulanger, Chakraborty, Tempe, Piechaczyk, & Bossis, 2021). To summarize, we use bioinformatics approaches to initiate from two sets of transcript sequencing data, execute mutual verification, perform dimensionality reduction techniques, merge the datasets, and perform functional enrichment analysis to obtain the key factor UHRF1 through an algorithm.

After we discovered the E3 ubiquitin protein ligase UHRF1, we went on to understand its effect on spinal cord injury(Citterio et al. 2004; Muto et al. 2002). By consulting the data, we discovered that it is a multi-domain protein, which acts as a key epigenetic regulator by linking DNA methylation and chromatin modification. It specifically recognizes and binds hemimethylated DNA at replication forks through its YDG domain and recruits DNMT1 methyltransferase to ensure the faithful propagation of DNA methylation patterns in DNA replication (Nady et al. 2011). In addition to its role in maintaining DNA methylation, it also plays a key role in chromatin modification (Unoki et al. 2004). This coincides with our conclusions obtained in the estimation analysis. It is enriched in pericentric heterochromatin, where it recruits different chromatin modifiers required for replication of this chromatin (Bostick et al. 2007). Also localized in heterochromatin regions, it may negatively regulate transcription by affecting DNA methylation and histone modifications (Sharif et al. 2007). Therefore, it is very likely to play a role in improving spinal cord injury by intervening in it.

Subsequently, UHRF1 was verified in vivo and in vitro by means of experiments familiar to our team. At the level of C57 mice, intervening with them can see a partial recovery of mouse motor function at an early stage. And our team's research on zinc for many years can also promote the same effect. It is a relatively safe and appropriate method to use trace elements which are very abundant in the human body for treatment. Therefore, our team has conducted research on the treatment of spinal cord injury with zinc for more than 10 years (Ge et al. 2021; Hu et al. 2021; Li et al. 2020). This undoubtedly provides a new way of thinking for us to explain the role of zinc. Subsequently, we performed immunofluorescence staining on mouse spinal cord tissue samples and found that ZnG could reduce the expression of UHRF1. Subsequently, the expression of UHRF1 in PC12 cells was tried by confocal staining, and the same result was obtained. So far, we have proved the key role of UHRF1 in the early stage of spinal cord injury, especially in the 3d stage, through the combination of biological information and experimental verification.

Finally, in the subsequent phase of our research, we also expect to combine basic experiments with more technical tools to explore new targets for the treatment of spinal cord injury. For the validation of UHRF1, we also hope to further explore the specific role of methylation in epigenetics and its upstream and downstream regulatory relationships. We expect to explore the story of UHRF1 at most time points after spinal cord injury. Of course, we will continue to introduce more molecular regulatory mechanisms of zinc and spinal cord injury in our follow-up work.

## Data Availability

Enquiries about data availability should be directed to the authors.
